# Revealing Nanoscale Solute‐Rich Clusters in Bulk Metallic Glasses by Atom Probe Tomography

**DOI:** 10.1002/smtd.202500980

**Published:** 2025-08-14

**Authors:** Keita Nomoto, Huma Bilal, Bosong Li, Bernd Gludovatz, Christoph Gammer, Anton Hohenwarter, Jürgen Eckert, Jamie J. Kruzic, Simon P. Ringer

**Affiliations:** ^1^ Australian Centre for Microscopy & Microanalysis and School of Aerospace, Mechanical and Mechatronic Engineering The University of Sydney Camperdown 2006 Australia; ^2^ School of Mechanical and Manufacturing Engineering University of New South Wales (UNSW Sydney) Sydney 2052 Australia; ^3^ Erich Schmid Institute of Materials Science Austrian Academy of Sciences Jahnstraße 12 Leoben 8700 Austria; ^4^ Department of Materials Science Chair of Materials Physics Montanuniversität Leoben Leoben 8700 Austria

**Keywords:** atom probe tomography, bulk metallic glass, solute‐rich cluster, structure‐property relationship

## Abstract

Bulk metallic glasses (BMGs) exhibit excellent mechanical properties due to their lack of long‐range atomic ordering. However, understanding their structure‐property relationships remains an unresolved challenge since traditional characterization methods have been unable to reveal the 3D nanostructures that control mechanical properties. In this study, a novel approach is developed that uses atom probe tomography (APT) cluster analysis to identify and visualize 3D nanoscale solute‐rich clusters in Zr‐based BMGs and quantify their size, composition, spatial distribution, and volume fraction. These results show that hardness variations in BMGs are driven by the volume fraction and distribution of solute‐rich clusters. By inputting these experimentally determined parameters into a model for ductile phase softening, the deformation mechanisms of BMGs are elucidated as being controlled by the solute‐rich clusters, and their possible relationship with topologically ordered short‐ and medium‐range ordered clusters is discussed. This methodological breakthrough in characterizing structure‐property relationships in metallic glasses is applicable to a wide range of multicomponent amorphous materials and is anticipated to enable major advances in glass science.

## Graphical abstract

This study presents a new methodology that resolves a longstanding challenge in multicomponent bulk metallic glasses by revealing 3D nanoscale solute‐rich clusters via atom probe tomography. The findings further establish direct correlations between solute‐rich clustering and local hardness, offering a powerful framework for investigating structure–property relationships in a wide range of metallic and non‐metallic multicomponent amorphous solids.

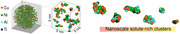

## 1 Introduction

BMGs are formed through rapid solidification processing from the liquid state, resulting in an amorphous structure where the atom positions lack long‐range structural order.^[^ ^1^ ^]^ While the amorphous structure provides BMGs with excellent properties (e.g., exceptional mechanical strength and elasticity,^[^ ^2^, ^3^ ^]^ superior soft magnetic properties,^[^ ^4^, ^5^ ^]^ etc.), establishing their structure‐property relationships has been an ongoing challenge due to the difficulties associated with observing their 3D nanostructures.^[^ ^6^, ^7^, ^8^, ^9^, ^10^, ^11^, ^12^ ^]^ The atomic structure of BMGs generally contains short‐range order (SRO) and medium‐range order (MRO) structural units that have long been studied in an averaged statistical manner using high‐energy X‐ray and/or neutron diffraction^[^ ^11^, ^12^, ^13^, ^14^, ^15^ ^]^ or by using various transmission electron microscopy (TEM) techniques.^[^ ^6^, ^7^, ^8^, ^16^, ^17^, ^18^, ^19^ ^]^ While such studies generally agree that MRO clusters form in a size range up to a few nanometers in diameter, their composition and 3D nanostructural arrangement remain essentially unknown. While atomic electron tomography has recently provided a 3D picture of the atomic arrangements inside individual SRO and MRO clusters in partially amorphous nanoparticles and thin films,^[^ ^16^, ^17^ ^]^ that technique is not applicable to BMG samples nor can it image the 3D nanostructural arrangement of ordered clusters/paracrystals that are thought to control the mechanical and magnetic properties of BMGs.^[^ ^6^, ^7^, ^8^, ^11^, ^12^ ^]^ To further advance the science of amorphous materials, it is imperative to develop characterization methods that can directly image the nanoscale structures that exist in BMGs.

APT has long been recognized as an ideal technique for nanoscale compositional analysis and imaging 3D nanostructural features in solids.^[^ ^20^ ^]^ However, the use of APT for studying fully amorphous metals has generally been limited to confirming nanoscale chemical homogeneity,^[^ ^6^, ^21^ ^]^ revealing phase separation,^[^ ^22^, ^23^, ^24^ ^]^ or determining nearest neighbor pair statistics.^[^ ^25^, ^26^, ^27^ ^]^ One limitation of APT is its inability to directly observe topological ordering (e.g., icosahedral or crystal‐like ordering), for which diffraction methods make an excellent complementary technique. In this study, we utilize APT and a cluster analysis approach to directly observe and quantify the 3D distribution, chemical composition, and volume fraction of nanoscale solute‐rich clusters in two Zr‐based BMGs. We then uncover the structure‐property relationships governed by these clusters by conducting site‐specific APT in regions with higher or lower hardness within both BMGs. Analysis of the separate hard and soft regions within BMG samples reveals that the cluster volume fraction and distribution control the local hardness. This investigation offers a whole new approach to studying structure‐property relationships for metallic glasses that should have wide implications for the study of amorphous inorganic solids.

## 2 Results

### 2.1 APT Cluster Analysis

**Figure 1:**
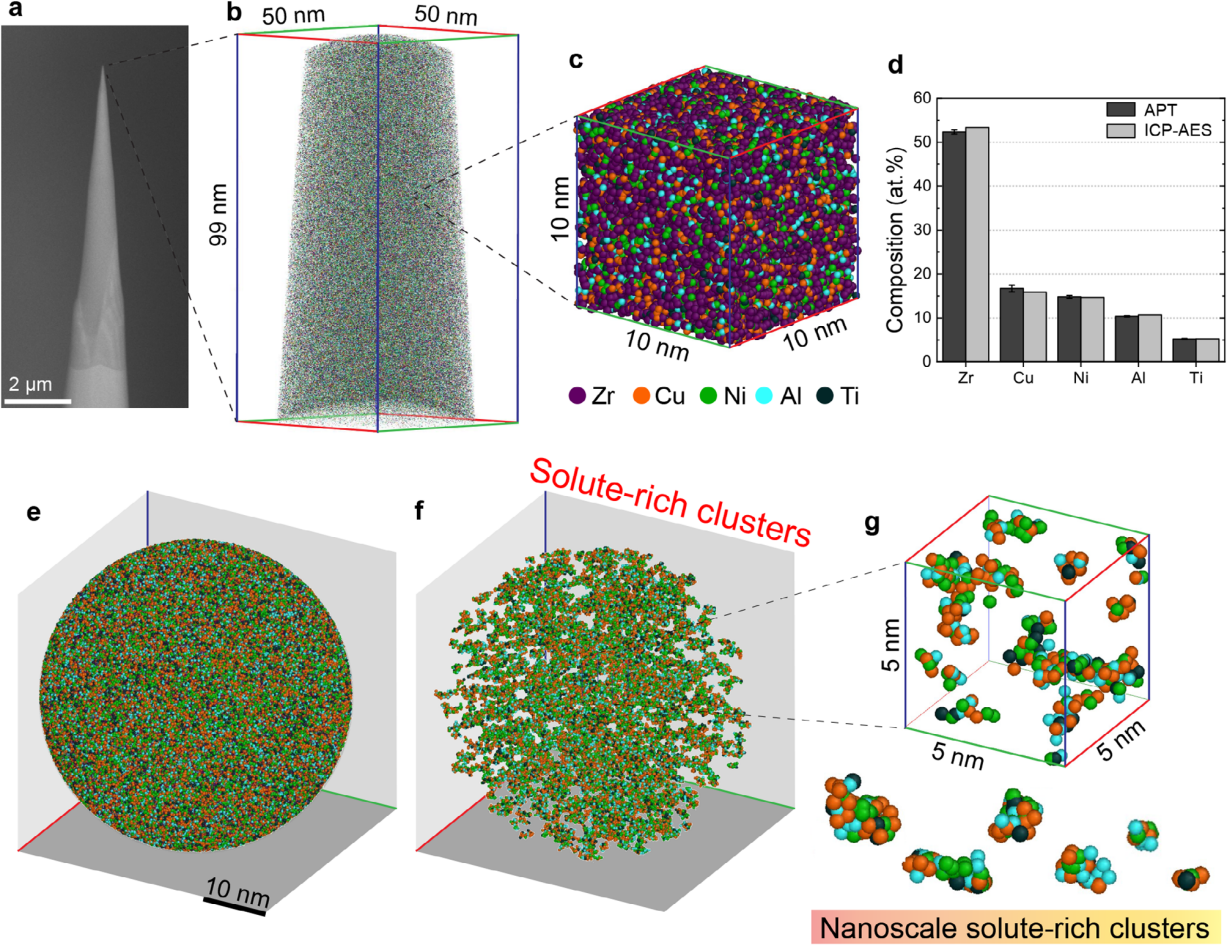
3D characterization of nanoscale solute‐rich clusters in Zr–Cu–Ni–Al–Ti BMG. a) Secondary electron micrograph of an APT specimen. b) Representative 3D APT reconstruction. c) Tomographic atom map extracted from a 10 nm × 10 nm × 10 nm cubic volume in (b), illustrating the distribution of Zr (purple), Cu (orange), Ni (green), Al (cyan), and Ti (dark green) atoms. d) Chemical composition analysis comparing APT and ICP‐AES results that demonstrates the compositional accuracy of APT relative to the bulk average composition. e) A region of interest displayed as a sphere showing the Cu, Ni, Al, and Ti atoms with Zr atoms excluded to enhance visual clarity. f) Visualization of solute‐rich clusters extracted from the region of interest shown in (e), highlighting their distribution. g) 3D representation of solute‐rich clusters extracted from (f), showcasing a range of sizes and morphologies at the nanoscale.

**Figure** **1**a–c showcases the multi‐scale APT methodology, ranging from the size of an APT specimen to tomographic atom maps for the Zr_52.5_Cu_17.9_Ni_14.6_Al_10_Ti_5_ (Zr–Cu–Ni–Al–Ti) BMG. In a 10 nm‐cube, the atoms appear randomly distributed to the eye, and the chemical composition determined by APT closely matches the bulk composition measured by inductively coupled plasma atomic emission spectroscopy (ICP‐AES), as shown in Figure 1d. Similarly, for the Zr_63.78_Cu_14.72_Ni_10_Al_10_Nb_1.5_ (Zr–Cu–Ni–Al–Nb) BMG, the bulk chemical compositions obtained by APT and ICP‐AES match (see Figure S1, Supporting Information). The cluster finding algorithm was utilized to identify and group clusters of atoms, distinguishing experimental APT data from random distributions, as described in the Experimental Section. Solute‐rich clusters are extracted from the amorphous structure (Figure 1e) and are visualized in three dimensions in Figure 1f. This facilitates the direct observation of nanoscale solute‐rich clusters (Figure 1g), enabling observations of their distribution and the quantification of their size, chemical composition, and volume fraction.

**Figure 2:**
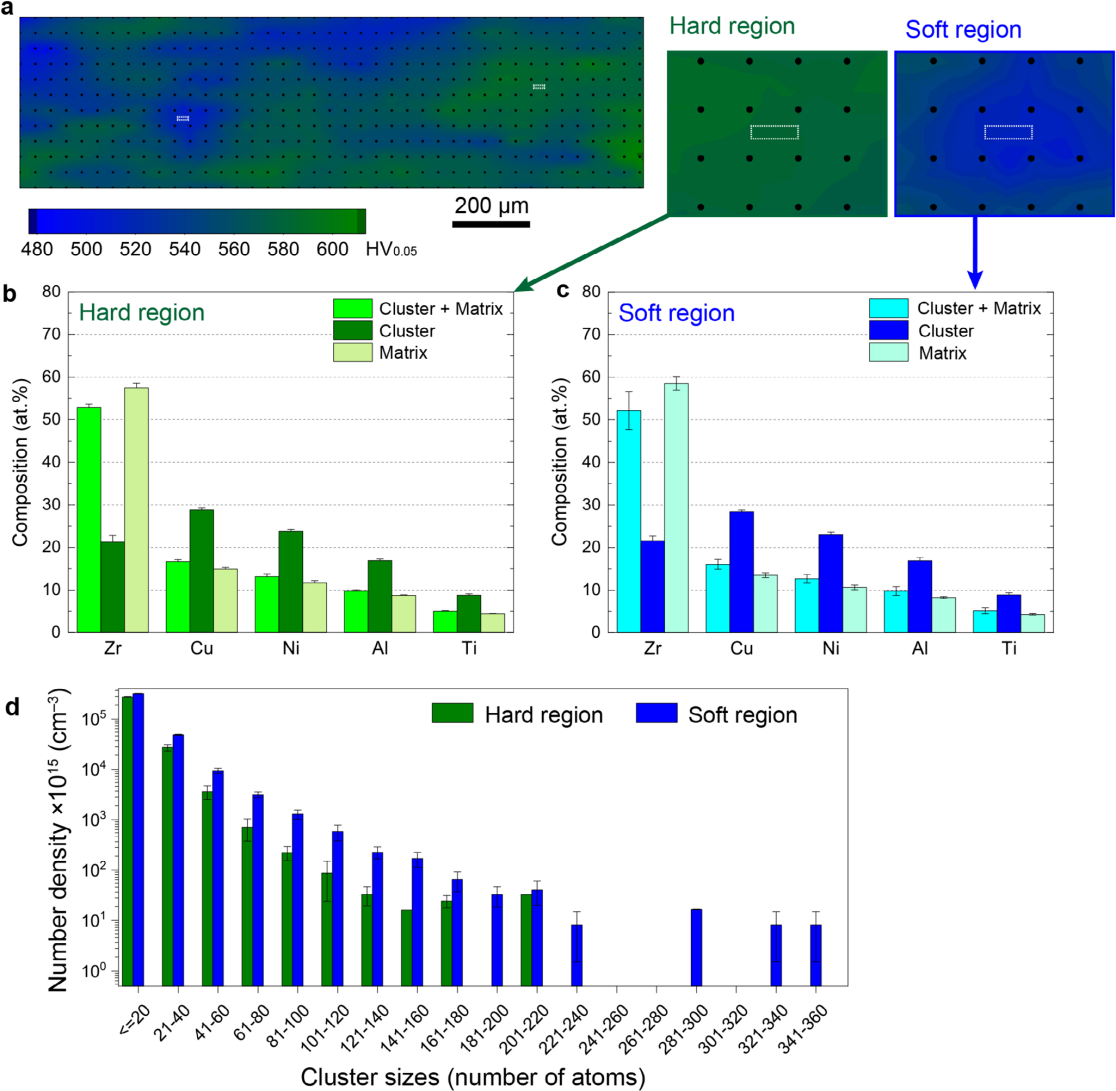
Chemical composition and number density of solute‐rich clusters. a) Microhardness map of the Zr‐Cu‐Ni‐Al‐Ti BMG, illustrating the distribution of hard (green) and soft (blue) regions. b,c) Chemical composition analysis within solute‐rich clusters and their surrounding matrix for both hard and soft regions, showing an enrichment of solute elements (Cu, Ni, Al, Ti) in the nanoscale clusters. d) Number density of the solute‐rich clusters derived from APT samples extracted from hard and soft regions in the BMG microstructure. The data were averaged from at least three samples for each region, and the error bars represent one standard deviation. The large solute‐rich clusters, comprising over 220 atoms, are only observed in the soft regions.

In many BMGs, structural heterogeneities in the form of hard and soft regions are distributed on a microstructural scale, as is shown in **Figure** **2**a and as has been reported in numerous studies.^[^ ^6^, ^7^, ^28^, ^29^, ^30^, ^31^, ^32^, ^33^ ^]^ APT specimens were lifted out using a focused ion beam (FIB) from regions between four indents of nearly identical hardness, where the average hardness values could clearly differentiate between relatively harder and softer regions using the methods described in refs. [6, 7]. Moreover, indents were spaced sufficiently such that FIB extraction occurred outside of the plastic zone of the surrounding indents.^[^ ^6^ ^]^ APT‐based cluster analysis was conducted on these areas of known hardness, and the chemical compositions within the solute‐rich clusters (Cluster), outside the clusters (Matrix), and total volume (Cluster + Matrix) were measured for both hard regions (Figure 2b) and soft regions (Figure 2c). In the Zr–Cu–Ni–Al–Ti BMG, the clusters are enriched in solute atoms (Cu, Ni, Al, Ti), with concentrations at approximately twice the nominal composition. However, the average chemical compositions of hard and soft regions are almost identical when the clusters and matrix are grouped together. The same trend has also been observed for the Zr–Cu–Ni–Al–Nb BMG (Figure S2a,b, Supporting Information).

Figure 2d compares the number density per volume of solute‐rich clusters in hard and soft regions. The average number densities across all size ranges of clusters in the soft region are significantly higher than those in the hard region. Notably, the maximum cluster size range reaches up to 360 atoms for the soft region, compared to a maximum of 220 atoms in the hard region. This trend is similar for the Zr–Cu–Ni–Al–Nb BMG (Figure S2c, Supporting Information). Figure S2d,e (Supporting Information) displays a classification of the morphology of the solute‐rich clusters, which are various but predominantly exhibit spherical and disk‐like shapes.

### 2.2 Structure‐Property Relationships

**Figure 3:**
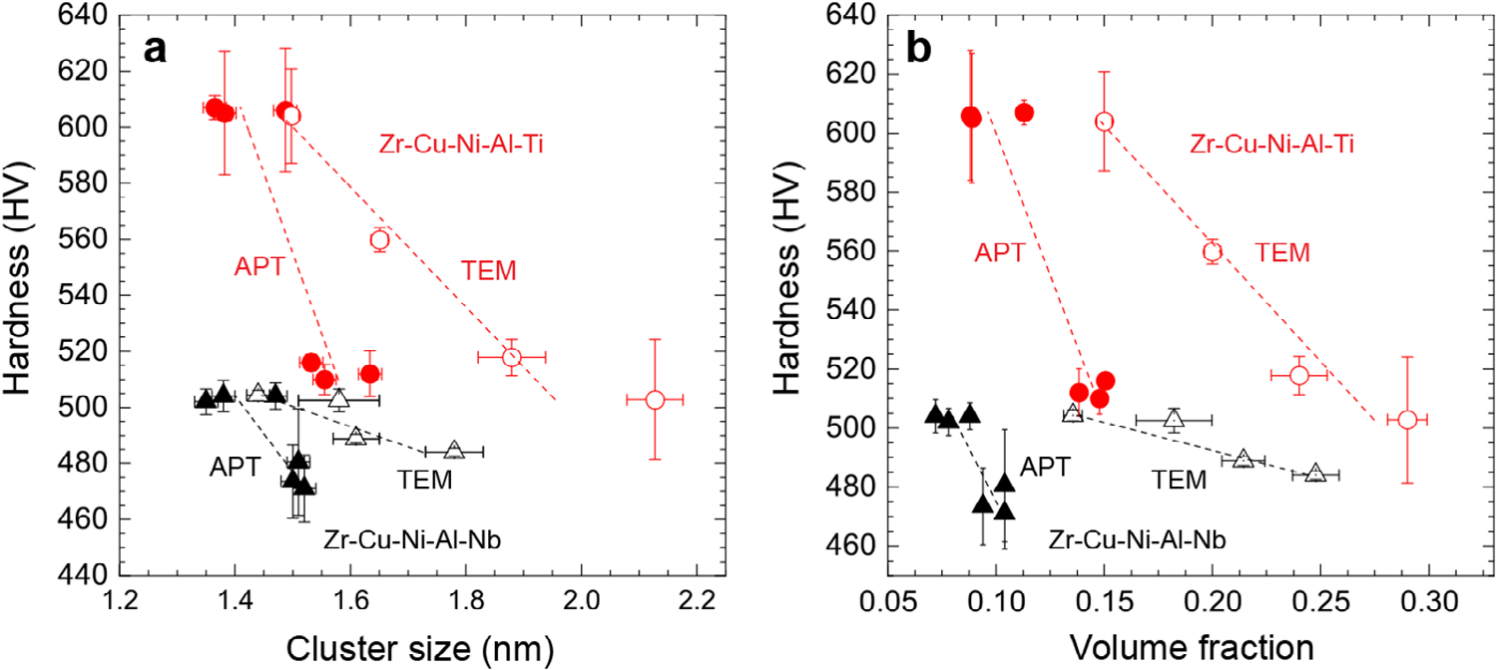
Correlation between hardness and cluster size and volume fraction for Zr–Cu–Ni–Al–Ti BMG and Zr–Cu–Ni–Al–Nb BMGs, based on APT and TEM methods. a) Hardness versus size of solute‐rich clusters from APT and MRO clusters from TEM, demonstrating an inverse relationship; as the size of either cluster type increases, the hardness decreases. b) Hardness versus volume fraction of solute‐rich clusters from APT and MRO clusters from TEM, illustrating that an increase in the volume fraction of either cluster type correlates with a decrease in hardness. The hardness data were averaged from regions between four indents, with error bars representing one standard deviation. The solute‐rich cluster size data were averaged from all clusters with a diameter larger than 1 nm, and the error bars indicate one standard deviation.

In our APT cluster analysis methodology, the number of atoms inside a cluster determines its size. To enable the development of structure‐property relationships for these BMGs, the APT cluster sizes were converted into a linear dimension. To facilitate this conversion, the clusters were assumed to be spheres, and their diameters were calculated from the sphere volumes as described in the Experimental Section. **Figure** **3** shows the hardness plotted as a function of cluster size and volume fraction derived from the APT‐based analyses for the Zr–Cu–Ni–Al–Nb BMG and Zr–Cu–Ni–Al–Ti BMG samples. For both BMG alloys, the average cluster size increases as the hardness decreases (Figure 3a). Similarly, the volume fraction of the clusters increases as the hardness decreases (Figure 3b). Statistically significant linear correlations between cluster size/volume fraction and hardness are found for both BMG compositions. Also shown in Figure 3 are results from previous TEM‐based studies of face centered cubic (FCC)‐like MRO clusters in the Zr–Cu–Ni–Al–Ti and Zr–Cu–Ni–Al–Nb BMGs used in this study.^[^ ^6^ ^]^ Those results revealed a linear trend of decreasing hardness with increasing MRO cluster size and volume fraction. A comparison of the results from these two different studies will follow in the Discussion section.

## 3 Discussion

### 3.1 Characteristics of the Solute‐Rich Clusters

The present work demonstrates the first direct experimental observations of the size distribution, chemical composition, volume fraction, and 3D spatial positioning of nanoscale solute‐rich clusters in multicomponent BMGs using APT. These data represent tens of thousands of clusters, ensuring statistical accuracy. Moreover, these clusters represent a form of chemical ordering in metallic glasses, and our APT results indicate the existence of various types of solute‐rich clusters, differing in size, composition, and morphology (Figures 1g and 2d; Figure S2d–e, Supporting Information). For example, individual clusters may be relatively more enriched in Cu, Ni, or Al, compared to the average values, especially for relatively smaller clusters with too few atoms to maintain the statistical average. Furthermore, the clusters have a range of sizes (Figures 1 and 2), suggesting that small clusters are interconnected to form nanoscale solute‐rich clusters by sharing atoms. The diameter of solute‐rich clusters was estimated from their volume to be ≈1.3–1.7 nm on average (Figure 3a), though their volume may be a more representative measure for describing the size of variable‐shaped clusters that are not always spherical. A key question that arises is whether these solute‐rich clusters exhibit topological ordering and whether they might be related to the topological SRO/MRO clusters observed by various diffraction and TEM‐based methods. Unfortunately, identifying the detailed topological arrangement of atoms within solute‐rich clusters directly via APT is so far impossible due to the limited spatial resolution of current atom probe techniques.^[^ ^34^ ^]^ Unlike the nearly perfect atomic arrangement in crystalline structures, the disordered atomic arrangement in amorphous structures excludes us from inferring the topological ordering from the known crystalline lattice. However, according to the well‐known efficient cluster packing model, topological ordering in multicomponent BMGs is mainly thought to consist of solute‐centered SRO clusters within a solvent matrix, which then aggregate to form MRO clusters.^[^ ^16^, ^35^ ^]^ Previous studies on these same two Zr‐based BMGs indicated that topological MRO clusters are likely composed of Zr_2_Ni, Zr_2_Cu, and/or ZrCuNi FCC‐like structures distributed within a less‐ordered glassy matrix.^[^ ^6^ ^]^ Furthermore, similar linear correlations of cluster size and volume fraction with local hardness are found for both the solute‐rich clusters revealed by APT and topological MRO clusters found by a previous TEM‐based analysis,^[^ ^6^ ^]^ as shown in Figure 3. The collective information suggests that the APT‐observed solute‐rich clusters may correspond to the topological MRO clusters identified by TEM; however, the direct one‐to‐one connection of these features is so far impossible. Nonetheless, the correlations of solute‐rich cluster size and volume fraction with local hardness suggest they play a significant role in the deformation process for these BMGs, which will be discussed in the next section.

### 3.2 Deformation Mechanisms in BMG

**Figure 4:**
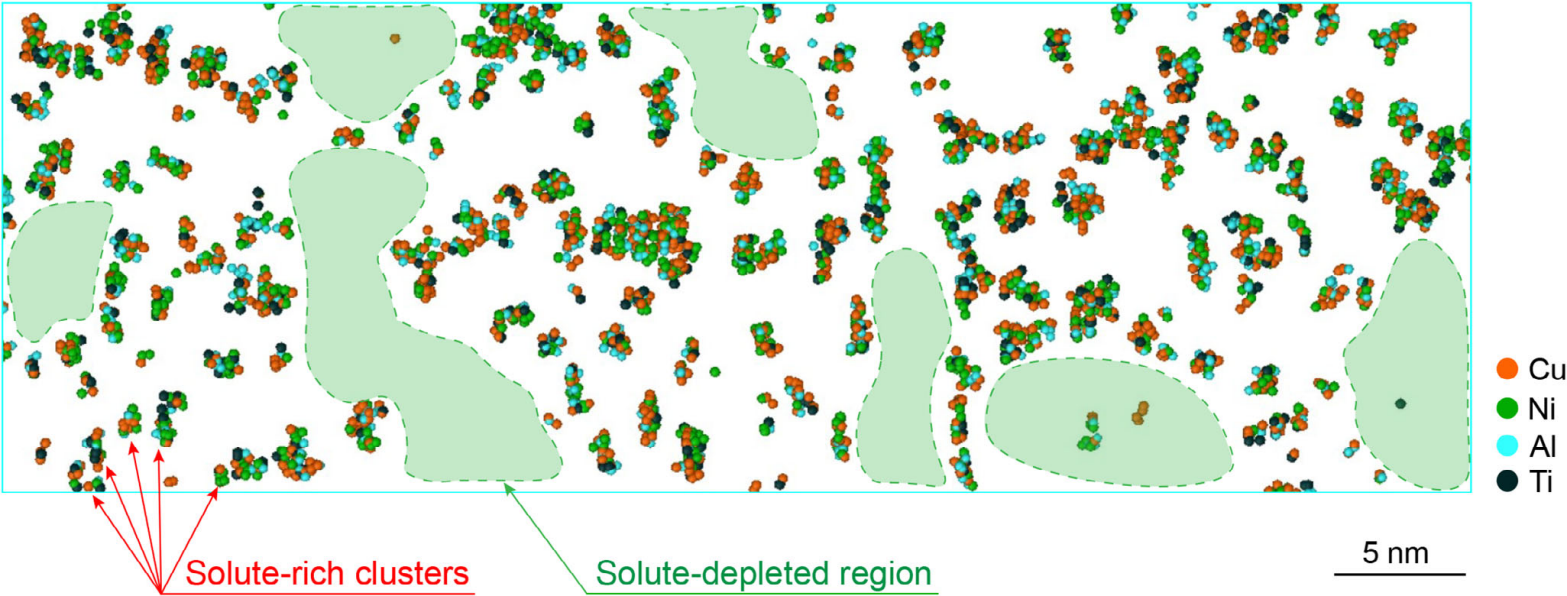
Role of solute‐rich clusters in deformation mechanisms of BMGs. A 2 nm thick slice extracted from the actual APT data, showing the real distribution of solute‐rich clusters in the Zr–Cu–Ni–Al–Ti BMG. Only solute‐rich clusters are visible in this figure with the matrix atoms removed. The green‐shaded areas indicate solute‐depleted regions. This variation in cluster distribution may indicate where deformation could initiate by the percolation of STZ activity at solute‐rich clusters that act as precursors to shear bands.

An increasing body of evidence suggests that locally soft spots, Eshelby‐like inclusions, and/or clusters of close‐packed atoms may initiate inelastic atomic displacements in a glassy matrix under strain.^[^ ^36^, ^37^, ^38^, ^39^, ^40^ ^]^ These displacements may connect and interact with neighboring regions, transferring stress to initiate the deformation of harder, neighboring sites to eventually form shear transformation zones (STZs) and nucleate shear bands.^[^ ^36^, ^41^ ^]^ The local atomic displacements may also generate free volume sites or nanocavities, as indicated by experimental results and molecular dynamics simulations.^[^ ^42^, ^43^ ^]^ Furthermore, shear band nucleation and propagation induce an increase in local temperature and diffusion kinetics, significantly changing the original atomic arrangement.^[^ ^7^, ^18^, ^44^, ^45^, ^46^ ^]^ **Figure** **4** shows a 2 nm thick volume slice extracted from the actual APT data and illustrates the heterogeneous distribution of the solute‐rich clusters, with the existence of solute‐rich and solute‐depleted regions. While these regions co‐exist within both the microscale hard and soft regions (Figure 2a), the observed correlation of increased solute‐rich clustering with lower hardness (Figure 3) suggests that these clusters act as soft spots in the structure that may trigger STZ activity.

The large‐scale observation of the solute‐rich cluster distribution shown in Figure 4 provides a picture of the original nanostructural features before shear transformations begin, offering valuable insights into where the deformation mechanisms might initiate in BMGs. The solute‐rich regions, where solute‐rich clusters are closely positioned, create a potential percolation pathway of STZ precursors, whereas the solute‐depleted regions (green shaded areas) would be less likely to initiate deformation.

To quantify this effect, we can use a model for ductile phase softening of dual‐phase metals,^[^ ^47^, ^48^ ^]^ which has also been applied to these same BMGs to understand the softening effect of topological MRO clusters.^[^ ^6^ ^]^ By assuming that hardness is related to yield stress by *H*_v_ ≈ 3𝜎_y_,^[^ ^49^, ^50^ ^]^ local yield strength values of 𝜎_y_ ≈ 1.54–1.65 GPa for the Zr‐Cu‐Ni‐Al‐Nb BMG and 1.66–1.98 GPa for the Zr‐Cu‐Ni‐Al‐Ti BMG can be deduced, which agree well with reported bulk yield strength values for these BMGs (Table S1, Supporting Information). Assuming that the softer nanoscale solute‐rich clusters yield first, then overall yielding of the BMG should occur when the following condition is met:^[^ ^47^ ^]^

(1) $$\frac{H_{v}}{3}=\sigma_{y}=\sigma_{y}^{m}-\sqrt{3}E^{m}\left(1-\beta \right)\gamma_{pl}\phi$$

In Equation (1), $\sigma_{y}^{m}$ and *E^m^* represent the yield strength and elastic modulus, respectively, of the harder BMG matrix, γ_*pl*_ is the plastic strain of nanoscale solute‐rich clusters at the point of overall composite yielding, and Φ is their volume fraction. 𝛽 is the material constant of Eshelby's S‐tensor for spherical inclusions, 𝛽 = 0.133(4 − 5ν^*m*^)/(1 − ν^*m*^), where ν^*m*^ is the Poisson's ratio for the BMG matrix.^[^ ^51^ ^]^ In this analysis, we have neglected stress concentrations due to elastic mismatch between the clusters and matrix since the elastic properties are assumed to be similar.

Based on Equation (1), the intercepts from the linear fits in Figure 3b are equal to 3$\sigma_{y}^{m}$ while the slopes are equal to $3\sqrt{3}E^{m}\left(1-\beta \right)\gamma_{pl}$. From the intercept values of the APT‐based cluster analysis (Table S2, Supporting Information), $\sigma_{y}^{m}$ is determined to be 1.90 and 2.49 GPa for the Zr‐Cu‐Ni‐Al‐Nb and Zr‐Cu‐Ni‐Al‐Ti BMGs, respectively. As expected, these values fall above the reported yield strengths for those BMGs since they only represent the harder matrix. The slope depends on elastic constants and the plastic shear strain sustained by the softer clusters prior to overall yielding, γ_*pl*_. Since the elastic constants are comparable for the two BMGs (Table S2, Supporting Information), γ_*pl*_ can be considered the main differentiating factor defining the different slopes for the two BMGs. Solving for γ_*pl*_ using the slope values shown in Table S2 (Supporting Information) gives values of 0.042 and 0.068 for the Zr‐Cu‐Ni‐Al‐Nb and Zr‐Cu‐Ni‐Al‐Ti BMGs, respectively. A detailed discrete STZ plasticity model for BMGs found in the literature has suggested that ductile behavior is associated with an STZ shear transformation strain magnitude of ≈0.05.^[^ ^40^ ^]^ The theoretical results agree well with the plastic strain values of 0.042 and 0.068 found for the Zr‐Cu‐Ni‐Al‐Nb and Zr‐Cu‐Ni‐Al‐Ti BMGs, respectively, based on the APT volume fraction results. Furthermore, both BMGs used in this study demonstrate extensive compression and bending ductility,^[^ ^29^, ^30^ ^]^ and thus values in the range of 0.042 and 0.068 are consistent with their macroscopic mechanical response. In this regard, the APT cluster analysis developed in this study provides a fundamentally new methodology for developing a detailed understanding of the structure‐property relationships for BMGs.

It is also important to mention that an increase in hardness is also generally associated with a decrease in free volume, as shown in refs. [29, 30, 52] for these two BMGs by using differential scanning calorimetry to measure the relaxation enthalpy. However, the APT approach used in this study does not have sufficient resolution to detect small differences in free volume, nor can it detect the location of free volume sites in the atomic structure of BMGs. Thus, there is still considerable work to be done to establish the full 3D atomic structure of metallic glasses and the detailed relationships between solute clustering, topological ordering, and free volume. The next section will discuss the possible connections between solute clustering and topological ordering.

### 3.3 Possible Connections Between Solute‐Rich Clusters and Topologically Ordered Clusters

Most previous work on atomic clustering in BMGs has focused on topological SRO and MRO revealed by various diffraction and TEM‐based methods, and such studies suggest that crystal‐like (e.g., FCC, HCP, BCC, or SC) clusters and polyhedral (e.g., icosahedral and pentagonal bipyramids) clusters often co‐exist.^[^ ^6^, ^16^, ^17^, ^19^, ^53^ ^]^ While the definition of medium‐range ordering is less agreed upon compared to that of short‐ or long‐range ordering,^[^ ^54^ ^]^ this inherent ambiguity may reflect the actual variability in the MRO clusters, and one should not expect to observe the same trends for very different types of MRO. For example, FCC‐like MRO clusters are expected to be much softer than icosahedral‐like clusters.^[^ ^55^, ^56^ ^]^ Furthermore, it has been proposed that FCC‐like MRO clusters may act as precursors to STZ activity that provide percolation pathways for deformation, and the correlation of FCC‐like clusters measured by TEM with BMG hardness can be seen in Figure 3.^[^ ^6^ ^]^ This trend of softening with increasing amounts of FCC‐like MRO is very similar to the case where soft FCC nanocrystals are formed in an unrelaxed BMG matrix to provide softening to the overall composite structure.^[^ ^55^, ^57^ ^]^

Although the relationship between hardness and the size or volume fraction of FCC‐like MRO clusters is similar to the relationship seen for the APT measured solute‐rich clusters, we observe differences in the average size and volume fraction of the clusters determined by the two methods. Direct comparisons of the data in Figure 3 show that the values determined by TEM are higher. When the model described by Equation (1) was applied to the topological MRO clusters measured by TEM, very similar $\sigma_{y}^{m}$ values of 1.80 and 2.28 GPa, respectively, were obtained for the two BMGs.^[^ ^6^ ^]^ However, the γ_*pl*_ values of 0.042 and 0.068 determined for the Zr‐Cu‐Ni‐Al‐Nb and Zr‐Cu‐Ni‐Al‐Ti BMGs for the solute‐rich clusters were considerably larger than reported for the topological MRO clusters determined by TEM (0.005 and 0.011, respectively). The differences result from the volume fractions of solute‐rich clusters measured in this study being smaller than the volume fractions of the MRO clusters measured by TEM. For these to be the same clusters, explanations for these differences are required, and some possible contributing factors are discussed as follows. One contributing factor is that the MRO cluster volume fractions derived from the nano‐beam electron diffraction (NBED) experiments were reported as a Bragg‐active volume fraction in the form of *A*_hkl_ × Φ, where *A*_hkl_ is a Bragg‐active fraction and Φ is the physical volume fraction,^[^ ^6^ ^]^ due to the difficulty in quantifying the *A*_hkl_ value accurately. The peaks in the variance profile appear at ≈3.6 and 4.3 nm^−1^, and if we assume FCC crystal‐like structures, these peaks correspond to {111} and {200} planes, respectively. *A*_111_ and *A*_200_ are then calculated at 0.4 and 0.2, respectively, assuming the diameter of the paracrystal is 1.5 nm. As mentioned above, ref. [6] used an *A*_hkl_ value of 0.4 to determine the volume fraction that went into Equation (1). If we instead sum the *A*_111_ and *A*_200_ contributions to get a value of 0.6, the deduced values for γ_*pl*_ increase to 0.007 and 0.016 for the Zr‐Cu‐Ni‐Al‐Nb and Zr‐Cu‐Ni‐Al‐Ti BMGs, respectively, which are slightly closer to the APT‐based analysis. Another consideration is that the fluctuation electron microscopy model for calculating volume fractions^[^ ^58^ ^]^ assumes that there is either diffraction or no diffraction when the paracrystal is in or out of the Bragg condition. However, in a real experiment, when paracrystals are slightly tilted away from the Bragg condition, they would still show reduced intensity, not immediately zero, which strongly reduces the variance, resulting in the overestimation of the volume fraction in the TEM‐based analysis.

Next, it is important to recognize that defining the edge of a solute‐rich cluster by APT cluster analysis is challenging as solvent (Zr) atoms exist on the surface of the solute‐rich clusters, and some of those atoms might be topologically associated to the cluster and causes diffraction, yet be ignored by the chemical clustering analysis used for the APT data. This would lead to an underestimation of the size of clusters in the APT results compared to TEM. Additionally, for the APT data, the solute‐rich clusters were detected via solute‐centered atoms, meaning that solvent (Zr)‐centered clusters were not measured in the APT analyses. Yet, it is expected that Zr‐centered clusters exist in the matrix, and they are likely to be icosahedral‐like clusters that are detected by NBED in the TEM analysis. This factor would lead to smaller volume fractions measured by APT compared to TEM since the latter would include both FCC‐like and icosahedral‐like clusters. Additionally, if solvent‐(Zr) atoms or clusters bridge multiple solute‐rich clusters, the connections and their extra volume would not be recognized by the APT analysis. Thus, even if one assumes that the FCC‐like MRO clusters observed by TEM are solute‐rich, these combined factors will lead to the volume fractions and sizes of solute‐rich clusters detected by APT being smaller than MRO clusters measured by TEM. Thus, while it is not straightforward to create a one‐to‐one correlation between the solute‐rich clusters measured by APT and the FCC‐like topological MRO clusters measured by TEM, there are rational reasons why their apparent sizes and volume fractions should deviate based on the limitations of each characterization method.

## 4 Conclusion

In conclusion, we revealed the compositional and volumetric characteristics of solute‐rich clusters in three dimensions for two Zr‐based BMGs using APT. Our findings have enabled us to develop quantitative relationships between the size and volume fraction of solute‐rich clusters and the local hardness in BMGs, while additionally providing new insights into the chemical and morphological nature of solute clustering in these BMGs. This new methodological approach not only deepens our understanding of the structure‐property relationships of BMGs but also paves the way for new studies to advance the science of a wide range of amorphous inorganic solids.

## 5 Experimental Section

### Sample Preparation

Two types of Zr‐based BMGs with nominal compositions of Zr_52.5_Cu_17.9_Ni_14.6_Al_10_Ti_5_ (at.%) and Zr_63.78_Cu_14.72_Ni_10_Al_10_Nb_1.5_ (at.%) were used in this study, and the samples came from identical batches as used in previous studies.^[^ ^6^, ^7^, ^29^, ^30^ ^]^ In those studies, as‐cast samples were examined along with annealed, cryogenically thermally cycled, cold rolled, and high‐pressure torsion (HPT) deformed samples to achieve a wide range of local hardness and to establish correlations between local hardness and MRO clusters. Since the well‐known efficient cluster packing model for BMGs suggests MRO clusters are likely to be solute‐centered,^[^ ^16^, ^35^ ^]^ in this study a subset of the previously studied samples was used to look for a similar correlation of solute‐rich clusters with local hardness using APT.

The Zr–Cu–Ni–Al–Ti BMG, supplied by Liquidmetal Technologies, USA, was produced from research‐grade plates with dimensions of 30 × 30 × 2.3 mm^3^. Severe plastic deformation by HPT was used to achieve a large variation of local hardness in the sample. A disk with ≈8 mm diameter and 1 mm thickness was cut by wire electrical discharge machining. The specimen was then annealed at 630 K for 2 h to relieve residual stress and achieve a uniform structural state across the sample. The disc was then subsequently deformed by HPT at room temperature for 1 rotation at 0.2 rotations per minute under an applied stress of ≈7.6 GPa. The fully amorphous structure of the BMG sample was confirmed by high resolution TEM both after annealing and after HPT in a previous study.^[^ ^6^ ^]^

The Zr–Cu–Ni–Al–Nb BMG was prepared as described in ref.[30] by suction copper mold casting in a 99.999% high purity argon atmosphere from master alloys that were prepared by arc‐melting in a Ti‐gettered 99.999% high purity argon environment. For compositional homogeneity, the master alloys were re‐melted at least four times prior to casting. Cast rectangular beams were annealed at a temperature of 473 K for 10 min in a flowing high purity nitrogen atmosphere to relieve residual stresses. Cryogenic thermal cycling was conducted by alternatingly immersing the sample into liquid nitrogen and boiling water for 1 min each, for a total of 120 cycles. Further details about the materials and thermo‐mechanical procedures can be found in previous studies.^[^ ^6^, ^30^ ^]^

### Microhardness

Microhardness maps were measured on polished cross‐sections of the BMG samples using a Struers Durascan‐80 automated hardness testing using a Vickers indenter with a load of 0.05 kg‐force and a dwell time of 10 s. To avoid interference between the plastic zones of the indentations, a spacing of 40 µm was used between each indentation as described in detail in ref. [30].

### APT

Site‐specific APT specimens were prepared using a standard lift‐out technique^[^ ^20^ ^]^ in a Zeiss Auriga FIB scanning electron microscope equipped with a Kleindiek micromanipulator system, both from Germany. In previously published TEM studies,^[^ ^6^, ^7^ ^]^ the FIB conditions were established to avoid sample damage or nanocrystallization by comparing FIB fabricated samples to TEM samples produced by electropolishing and ion milling. To minimize potential ion beam‐induced damage, the ion beam incident angle was kept at ≈0 degrees during annular milling, a protective platinum cap layer was applied, and the final milling step was performed at a reduced acceleration voltage of 5 kV. APT measurements were performed using a CAMECA LEAP4000XSi atom probe equipped with a 355 nm ultraviolet laser. The experimental parameters were selected based on a previous study^[^ ^21^ ^]^ to achieve high quality mass spectra, including mass resolution, signal‐to‐thermal tail ratio, and overlapped peak ratio, to ensure accurate chemical composition data. The measurements were conducted at a temperature of 40 K within an analysis chamber pressure range of 10^−11^ to 10^−12^ Torr. A laser power of 75 pJ with a detection rate of 3% was used for the Zr–Cu–Ni–Al–Ti BMG, and a laser power of 100 pJ with a detection rate of 6% was used for the Zr–Cu–Ni–Al–Nb BMG. A laser pulse repetition rate of 160 kHz was used for both samples. Further details of the parameter study are described in ref.[21].

Reconstruction of the APT data was carried out using IVAS 3.6.8 software. A customized ranging approach (peak‐based ranging) was used throughout the current study to maintain the quality and robustness of the APT reconstructed data.^[^ ^21^ ^]^ The detector efficiency of the instrument had previously been determined to be ≈57%^[^ ^34^ ^]^ and was applied to the reconstruction algorithm. Although this detector efficiency cannot detect all clusters, its effect is uniform and random across the dataset. Therefore, as long as the same methodology is applied, the cluster distribution remains comparable. An image compression factor, *ξ*, of 1.5 and a field factor, *k_f_*, of 3.4 were determined using the method described by Loi et al.,^[^ ^59^ ^]^ according to the following equations:

(2) $$\xi =\left(c_{1}D+c_{2}\right)T+c_{3}D+c_{4}$$

(3) $$k_{f}=\left(1-\frac{1}{b_{1}V+b_{2}}\right)\xi^{3}-e^{-\left(b_{3}V+b_{4}\right)}+b_{5}$$

where *D* is the aperture diameter of 50 µm, *T* is the distance between the specimen tip and the aperture of 45 µm, and *c*_1_ to *c*_4_ and b_1_ to b_5_ are constants as follows: For *ξ =* 1.5, c_1_ = ‐9.286 × 10^−6^, c_2_ = 5.589 × 10^−3^, c_3_ = −4.643 × 10^−4^, and c_4_ = 1.499. For *k_f_* = 3.4 at a specimen voltage *V* of 7000, b_1_ = 0.3721, b_2_ = 1.3030, b_3_ = 0.2767, b_4_ = 3.4220, and b_5_ = 0.0670.

An average evaporation field, *F*, of 29 V nm^−1^ was calculated based on the evaporation field of the main element (Zr) and other solute elements with an evaporation field higher than the main element, and their molar fraction, *X*, as follows:^[^ ^21^ ^]^

(4) $$F=\sum \left(X_{\left(1-X_{\mathrm{Cu}+\mathrm{Ni}+\mathrm{Al}+\mathrm{Ti}\left(\mathrm{Nb}\right)}\right)}.F_{\mathrm{Zr}}+X_{\mathrm{Cu}}.F_{\mathrm{Cu}}+X_{\mathrm{Ni}}.F_{\mathrm{Ni}}+X_{\mathrm{Al}}.F_{\mathrm{Al}}+X_{\mathrm{Ti}\left(\mathrm{Nb}\right)}.F_{\mathrm{Ti}\left(\mathrm{Nb}\right)}\right)$$

The evaporation field values of the elements in Zr, Cu, Ni, Al, Ti and Nb were 28, 30, 35, 19, 26, and 37 V nm^−1^, respectively.

### Cluster Analysis

Cluster analysis of the APT data was first conducted using the IVAS software cluster analysis algorithm to determine the chemical composition of clusters based on their sizes. To ensure data reliability, the region of interest for the cluster analysis was selected from the core of the APT specimen, at least 10 nm away from the reconstructed surface, thereby excluding potentially ion beam‐induced damaged surface regions. The *K^th^* order nearest neighbor (NN) distributions and a maximum distance separating two solutes belonging to a cluster, *d*_max_, were used to describe the distance between ‘*K*’ atoms in the reconstruction. To determine the *K* value, a heuristic density‐based cluster finding algorithm (DBSCAN or KNN maximum separation) developed by Marceau et al.^[^ ^60^ ^]^ was used. The KNN approach is an empirical method for identifying clusters, comparing the difference between experimental and random cluster frequencies over a range of NN distances (*d*_max_). The *K* value was varied from 1 to 10, and this approach demonstrated a distinct difference between experimental and random cases of atomic distributions with 5NN (representative curves are plotted in Figure S3, Supporting Information) across all the datasets used in this study. Thus, *K* = 5 was selected for cluster analysis. The 5NN frequency histograms for the experimental and random data were compared using a custom MATLAB script, and *d*_max_ was selected as the distance of the largest difference between the experimental and random data. A minimum cluster size, *N*_min_, was selected as 6 atoms, which equals to *K *+ 1, as this represents the smallest number of atoms required to form a cluster. Cu, Ni, Al and Ti were chosen for the Zr–Cu–Ni–Al–Ti BMG, whereas Cu, Ni, Al and Nb were selected for the Zr–Cu–Ni–Al–Nb BMG, as the solutes used for identifying clusters in the present study. However, the sum of solute and solvent atoms was used when counting the number of atoms in a cluster. In order to compare the APT cluster size with other microscopy techniques, the clusters were converted to linear measurements, such as the diameter or radius of the clusters, assuming the clusters are spherical. The number density of clusters, *N_v_*, is the concentration of clusters in a given volume and is represented by the following equations:

(5) $$N_{v}=\frac{\left(N_{cluster}/0.57\right)}{V_{cluster}}$$

where *N*_cluster_ is the number of atoms in a cluster, *V*_cluster_ is the volume of a cluster, and the constant 0.57 accounts for the APT's detector efficiency (57%). *V*_cluster_ and *N*_v_ are defined using the following relations:

(6) $$V_{cluster}=\frac{4}{3}\pi {\left(\frac{d}{2}\right)}^{3}$$

(7) $$N_{v}=\frac{\sum \left(X_{Zr}.\rho_{Zr}+X_{Cu}.\rho_{Cu}+X_{Ni}.\rho_{Ni}+X_{Al}.\rho_{Al}+X_{Ti\left(Nb\right)}.\rho_{Ti\left(Nb\right)}\right)N_{A}}{\sum \left(X_{Zr}.m_{Zr}+X_{Cu}.m_{Cu}+X_{Ni}.m_{Ni}+X_{Al}.m_{Al}+X_{Ti\left(Nb\right)}.m_{Ti\left(Nb\right)}\right)N_{A}}10^{-21}nm^{3}$$

where *d* is the diameter of a cluster (nm), *X* is the molar fraction of the elements inside a cluster, *ρ* is the atomic density of the element (g nm^−3^), *N*_A_ is Avogadro's number (6.022 × 10^23^ atoms mol^−1^), and *m* is the molar mass of the elements inside the cluster (g mol^−1^). Substituting *N*_v_ and *V*_cluster_ from Equations (6) and (7) into Equation (5), the following equation was obtained:

(8) $$\begin{array}{ccc} & & \frac{\sum \left(X_{Zr}.\rho_{Zr}+X_{Cu}.\rho_{Cu}+X_{Ni}.\rho_{Ni}+X_{Al}.\rho_{Al}+X_{Ti\left(Nb\right)}.\rho_{Ti\left(Nb\right)}\right)N_{A}}{\sum \left(X_{Zr}.m_{Zr}+X_{Cu}.m_{Cu}+X_{Ni}.m_{Ni}+X_{Al}.m_{Al}+X_{Ti\left(Nb\right)}.m_{Ti\left(Nb\right)}\right)N_{A}}10^{-21}\\ & & =\frac{\left(N_{cluster}/0.57\right)}{\frac{4}{3}\pi {\left(\frac{d}{2}\right)}^{3}} \end{array}$$

Therefore, the diameter *d* can be calculated as:

(9) $$d={\left(\frac{\left(N_{cluster}/0.57\right)}{N_{v}}\times \frac{6}{\pi }\right)}^{1/3}$$

Finally, the morphology of the clusters was determined by calculating the oblateness and aspect ratio of the best‐fitted ellipsoid for each cluster.^[^ ^20^, ^61^ ^]^ Given the principal distances *L*_1_, *L*_2_, and *L*_3_ (*L*_1_> *L*_2_> *L*_3_), the shape of clusters mapped using the aspect ratio (*L*_2_/*L*_1_) and oblateness (*L*_3_/*L*_2_). The map was divided into four quadrants to categorize the clusters as disc, sphere, lath, or rod.

## Conflict of Interest

The authors declare no conflict of interest.

## Supporting information



Supporting Information

## Data Availability

The data that support the findings of this study are available from the corresponding author upon reasonable request.
