# Supplementary material for: Revealing Nanoscale Solute‐Rich Clusters in Bulk Metallic Glasses by Atom Probe Tomography
Source: Small Methods. 2025 Aug 14;9(9):e00980. doi: 10.1002/smtd.202500980 (PMC12464803; doi:10.1002/smtd.202500980)
Supplement: Supplementary file 1 — Supporting Information [file SMTD-9-e00980-s001.pdf]

small methods

Supporting Information

for *Small Methods*, DOI 10.1002/smtd.202500980

Revealing Nanoscale Solute-Rich Clusters in Bulk Metallic Glasses by Atom Probe Tomography

Keita Nomoto, Huma Bilal, Bosong Li, Bernd Gludovatz, Christoph Gammer, Anton Hohenwarter, Jürgen Eckert, Jamie J. Kruzic and Simon P. Ringer**

Supporting Information for

Revealing nanoscale solute-rich clusters in bulk metallic glasses by atom probe tomography

K. Nomoto[1*], H. Bilal[1*], B.S. Li[2], B. Gludovatz[2], C. Gammer[3], A. Hohenwarter[4], J. Eckert[3,4], J.J. Kruzic[2], S.P. Ringer[1]

[1]The University of Sydney, Australian Centre for Microscopy & Microanalysis, and School of Aerospace, Mechanical and Mechatronic Engineering, Australia

[2]School of Mechanical and Manufacturing Engineering, University of New South Wales (UNSW Sydney), Australia

[3]Erich Schmid Institute of Materials Science, Austrian Academy of Sciences, Austria

[4]Department of Materials Science, Chair of Materials Physics, Montanuniversität Leoben, Austria

[*]Equal contribution

Corresponding authors.
j.kruzic@unsw.edu.au (J.J. Kruzic), simon.ringer@sydney.edu.au (S.P. Ringer)

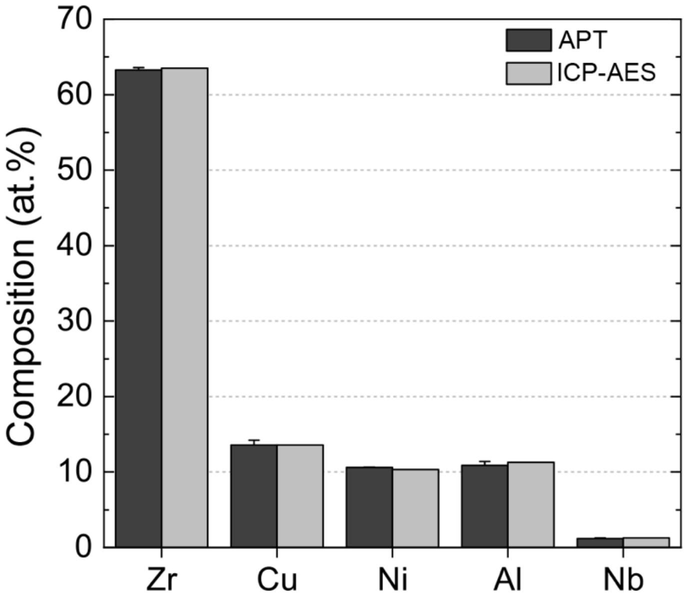

Figure S1. Chemical composition analysis comparing APT and ICP-AES. *This graph demonstrates the compositional accuracy of APT relative to the bulk average composition of the Zr–Cu–Ni–Al–Nb BMG.*

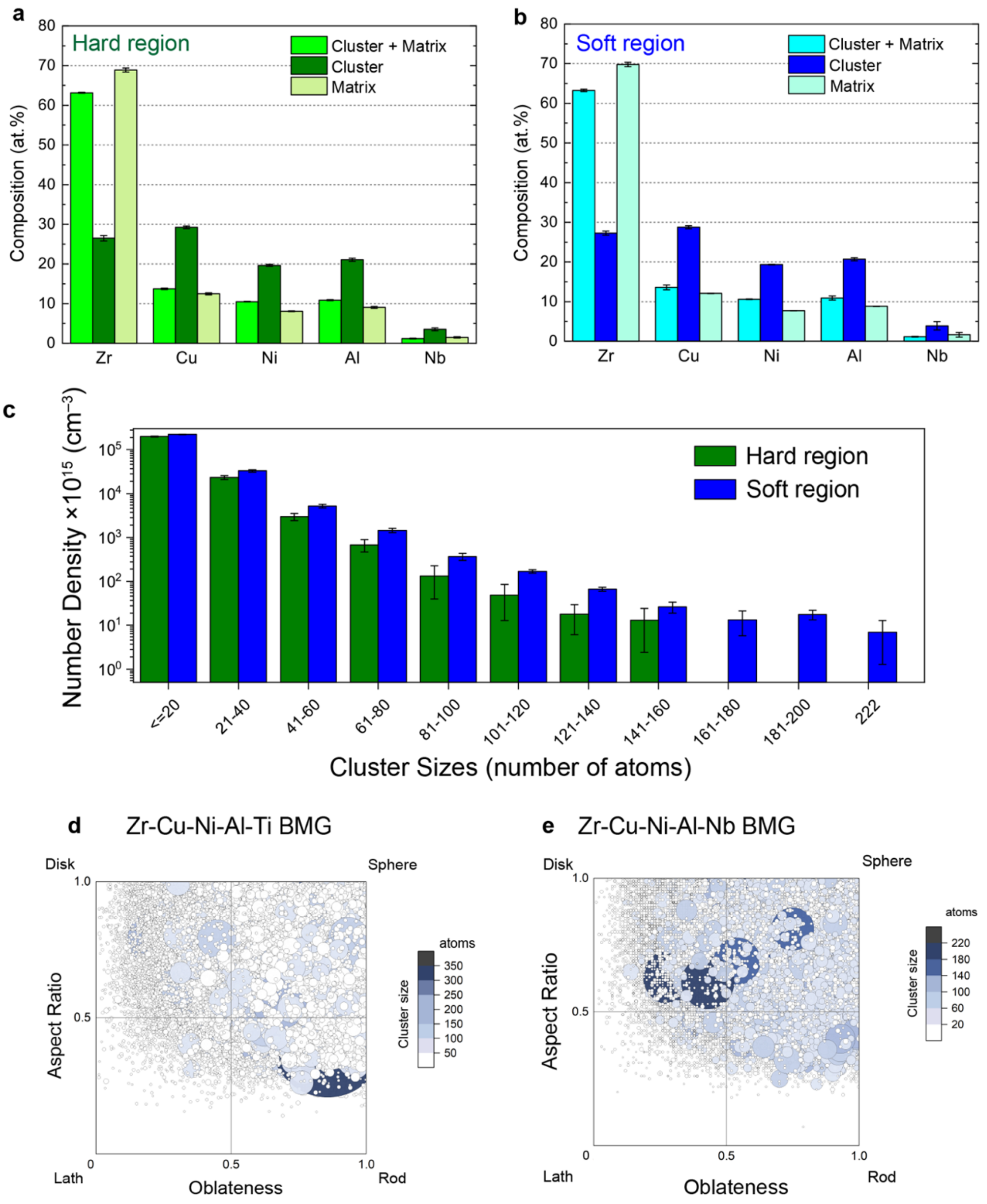

Figure S2. Chemical composition, number density, and morphologies of solute-rich clusters of the Zr–Cu–Ni–Al–Nb BMG. *(a, b) Chemical composition analysis within solute-rich clusters and their surrounding matrix for both hard and soft regions, showing an enrichment of solute elements (Cu, Ni, Al, Nb) in the nanoscale clusters. (c) Number density of the solute-rich clusters derived from APT samples extracted from hard and soft regions in the BMG microstructure. The data are averaged from at least three samples for each region, and the error bars represent one standard deviation. The largest solute-rich clusters comprising over 160 atoms are only observed in the soft regions. (d, e) Morphological classification of the solute-rich clusters in a soft region of Zr–Cu–Ni–Al–Ti and Zr–Cu–Ni–Al–Nb BMGs into four types (sphere, disk, lath and rod). The cluster sizes are indicated by the color scale and sizes of data point markers.*

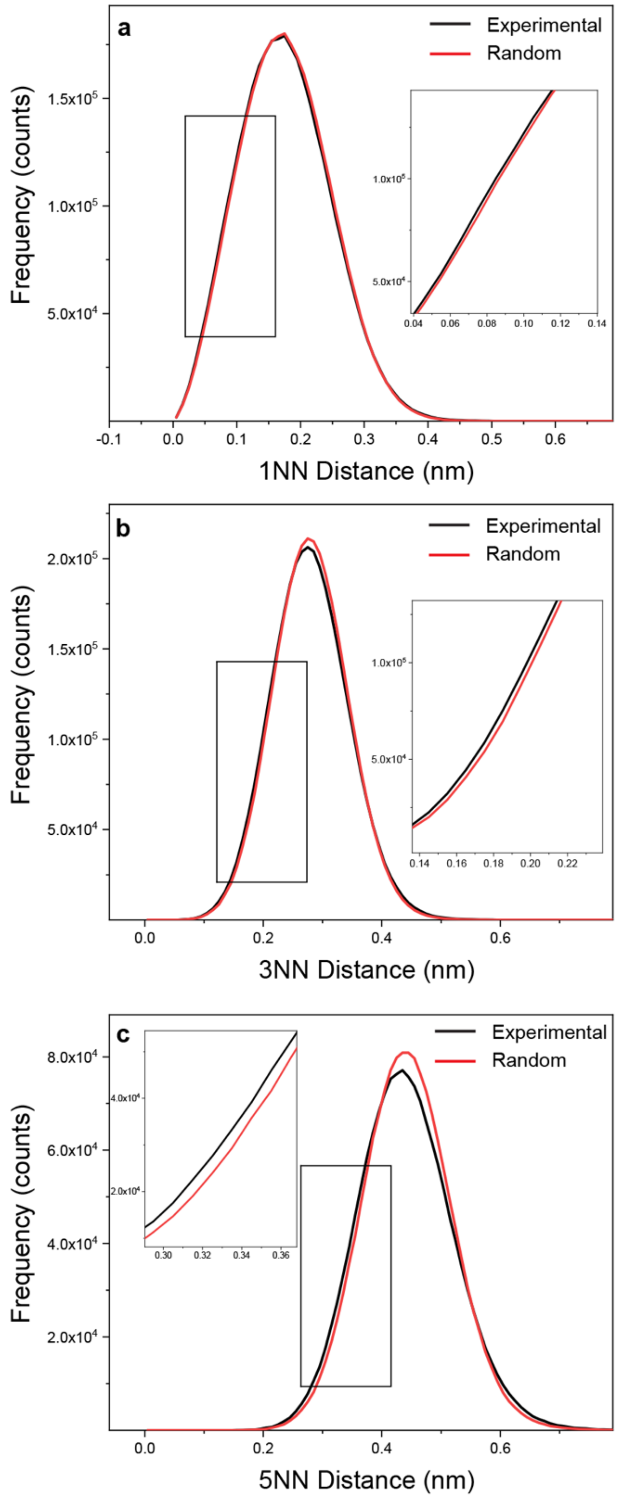

Figure S3. Nearest neighbor (NN) histogram *for (a) 1NN, (b) 3NN and (c) 5NN for both experimental and random datasets. The insets demonstrate the difference between the experimental and the random data for the left side of the curve. This demonstrates the distinct difference between experimental and random results with 5NN compared to lesser NNs.*

Table S1. Mechanical Properties of $Zr_{63.78}Cu_{14.72}Ni_{10}Al_{10}Nb_{1.5}$ and $Zr_{52.5}Cu_{17.9}Ni_{14.6}Al_{10}Ti_5$ taken from the literature. *Literature reference numbers are given in square brackets.*

BMG Composition (at.%)	Young's Modulus, E (GPa)	Poisson's Ratio, ν	β	Yield Strength, σ_y (GPa)
$Zr_{63.78}Cu_{14.72}Ni_{10}Al_{10}Nb_{1.5}$	82.1 [1]	0.390 [1]	0.448	1.50 – 1.80 [2, 3]
$Zr_{52.5}Cu_{17.9}Ni_{14.6}Al_{10}Ti_5$	85.6 [4]	0.375 [4]	0.453	1.65 – 1.85 [5-7]

Table S2. Summary of linear fit parameters for the plots based on the APT data in Fig. 3b.

BMG Composition (at.%)	Slope	Intercept	Correlation coefficient, R^2
$Zr_{63.78}Cu_{14.72}Ni_{10}Al_{10}Nb_{1.5}$	−3.30 GPa	1.90 GPa	0.72
$Zr_{52.5}Cu_{17.9}Ni_{14.6}Al_{10}Ti_{5}$	−5.48 GPa	2.49 GPa	0.87

References

1. Xie, S. H.; Kruzic, J. J., *J. Alloys Compd.* **2017,** *694*, 1109-1120.
2. Xie, S. H.; Tu, X. M.; Kruzic, J. J., *J. Alloys Compd.* **2018,** *735*, 1576-1581.
3. Xie, S. H.; Zeng, X. R.; Qian, H. X., *J. Alloys Compd.* **2009,** *480* (2), L37-L40.
4. Schneibel, J. H.; Horton, J. A.; Munroe, P. R., *Metall. Mater. Trans. A-Phys. Metall. Mater. Sci.* **2001,** *32* (11), 2819-2825.
5. Liu, C. T.; Heatherly, L.; Easton, D. S.; Carmichael, C. A.; Schneibel, J. H.; Chen, C. H.; Wright, J. L.; Yoo, M. H.; Horton, J. A.; Inoue, A., *Metal. Mater. Trans. A* **1998,** *29* (7), 1811-1820.
6. Morrison, M. L.; Buchanan, R. A.; Liaw, P. K.; Green, B. A.; Wang, G. Y.; Liu, C.; Horton, J. A., *Mater. Sci. Eng. A-Struct. Mater. Prop. Microstruct. Process.* **2007,** *467* (1-2), 190-197.
7. Zhang, Z. F.; Eckert, J.; Schultz, L., *Metall. Mater. Trans. A-Phys. Metall. Mater. Sci.* **2004,** *35A* (11), 3489-3498.
